# Preparation of Sodalite and Faujasite Clay Composite Membranes and Their Utilization in the Decontamination of Dye Effluents

**DOI:** 10.3390/membranes12010012

**Published:** 2021-12-23

**Authors:** Abderrazek El-kordy, Abdelaziz Elgamouz, El Mokhtar Lemdek, Najib Tijani, Salman S. Alharthi, Abdel-Nasser Kawde, Ihsan Shehadi

**Affiliations:** 1 Laboratory of Materials, Membranes, and Nanotechnology, Department of Chemistry, Faculty of Sciences, Moulay Ismail University, PB 11201, Zitoune, P.O. Box 11201, Meknes 50050, Morocco; abderrazekelkordy@gmail.com (A.E.-k.); lemdek@gmail.com (E.M.L.); najibtij@gmail.com (N.T.); 2 Pure and Applied Chemistry Group, Department of Chemistry, College of Sciences, University of Sharjah, Sharjah P.O. Box 27272, United Arab Emirates; akawde@sharjah.ac.ae (A.-N.K.); ishehadi@sharjah.ac.ae (I.S.); 3 Department of Chemistry, College of Science, Taif University, P.O. Box 11099, Taif 21944, Saudi Arabia; s.a.alharthi@tu.edu.sa

**Keywords:** sodalite, faujasite, clay membranes, zeolite membranes, ceramic membranes, methyl orange, retention, size exclusion, charge repulsion

## Abstract

The present work describes the deposition of two zeolite films, sodalite and faujasite, by the hydrothermal method to tune the mesopores of clay support, which are prepared from a widely available clay depot from the central region of Morocco (Midelt). The clay supports were prepared by a powder metallurgy method from different granulometries with activated carbon as a porosity agent, using uniaxial compression followed by a sintering process. The 160 µm ≤ Φ ≤ 250 µm support showed the highest water flux compared to the supports made from smaller granulometries with a minimum water flux of 1405 L.m^−2^·h^−1^ after a working time of 2 h and 90 min. This support was chosen for the deposition of sodalite (SOM) and faujasite (FAM) zeolite membranes. The X-ray diffraction of sodalite and faujasite showed that they were well crystallized, and the obtained spectra corresponded well with the sought phases. Such findings were confirmed by the SEM analysis, which showed that SOM was crystalized as fine particles while the FAM micrographs showed the existence of crystals with an average size ranging from 0.53 µm to 1.8 µm with a bipyramidal shape and a square or Cubo octahedral base. Nitrogen adsorption analysis showed that the pore sizes of the supports got narrowed to 2.28 nm after deposition of sodalite and faujasite. The efficiencies of SOM and FAM membranes were evaluated by filtration tests of solutions containing methyl orange (MO) using a flow loop, which were developed for dead-end filtration. The retention of methylene orange (MO) followed the order: SOM > FAM > 160 µm ≤ Φ ≤ 250 µm clay support with 55%, 48% and 35%, respectively. Size exclusion was the predominant mechanism of filtration of MO through SOM, FAM, and the support. However, the charge repulsion between the surface of the membrane and the negatively charged MO have not been ruled out. The point of zero charge (pzc) of the clay support, SOM and FAM membrane were pHpzc = 9.4, pHpzc = 10.6, and pHpzc = 11.4, respectively. Filtrations of MO were carried out between pH = 5.5 and pH = 6.5, which indicated that the surface of the membranes was positively charged while MO was negatively charged. The interaction of MO with the membranes might have happened through its vertical geometry.

## 1. Introduction

Water sources including seas, rivers, clouds (rain, ice, and snow), and underground are sources of pure water, water is pure by itself, and it is used to extract, purify, and solubilize many substances, it won the name of universal solvent due to its high polarity and its presence in every aspect of life. The physical characteristics of water could be changed either by presence of nonpolluting agents that are instigated from its origin such as rocks, sand, clay, and algal blooms formed because of water stagnation, or harmful pollutants originated from human activities. As a result, getting pure water has become a serious challenge and thus water sources must be protected [1]. In addition, drought resulted from climate change, and the increase of the industries’ installation are increasingly threatening water sources. Before the industrial revolution, natural dyes were largely in use, they were extracted from natural resources, such as insects, flowering plants, roots, and vegetables. However, with the increasing demand for dyes, many industries have become more dependent on synthetic dyes that provide a fast coloration process, and are more soluble and easily absorbable and versatile compared to natural dyes [2]. Textile is one of the industries that are using large amounts of water during their processes, releasing extensive amounts of synthetic dyes in the wastewater, hence contaminating water resources and land. Other industries such as printing, cosmetics, pharmaceutical, food packaging, and processing, are also dye polluting, but to a lesser extent compared to textile [3]. More than 15% of the dyes used in the textile industry are released to the environment without binding to the fibers. These are mixed with other organic and inorganic additives that are used to improve the adsorption of dyes on the fabrics [4]. Such chemicals usually end up in soils and surfaces [5]. Various research in the field of treatment of liquid effluents were implemented such as adsorption, flocculation, coagulation, and reverse osmosis [6]. Complementary processes of treatment, such as membrane’s filtration and advanced oxidation, are currently being developed for the wastewater treatment from some micropollutants like the dyes. Various ceramic membranes are fabricated from different materials [7,8], notably aluminum oxides, silicon carbides, zirconia, titanium oxide [9,10], and vitreous materials [11]. These membranes can be used for the filtration of liquids and gases [7]. Flat ceramic membranes and tubular clay-based have been used due to their parameters of supplementary performance such as the flow and the efficiency [12,13,14]. Different layers have been deposited on membrane supports (oxides, zeolites, and graphite), to obtain asymmetric membranes presenting the mechanical resistance and the thermal shocks resistance are desirable [15,16]. The main factors to take into account when using membranes in water filtration are the porosity, pores’ size, pores’ distribution, superficial charge, and the degree of hydrophobicity [17,18]. This research work is oriented towards the depollution of wastewater rich in dyes using clay/zeolite composite mineral membranes. The structural composition of aluminosilicates in the clays and zeolites are complementary since, in the clays, the sequences of silicon-oxygen tetrahedron and aluminum-octahedron form sheets while they form cage frames in zeolites. These sequence permits their use in ultrafiltration and nanofiltration [19,20]. Therefore, in the present research work, clay/zeolite ceramic membranes have been developed with the hope to surpass existing polymer membranes in the decontamination of the liquid effluents. The produced membranes are characterized by different techniques FTIR, XRD, BET, and surface charge before they are used in the removal of methyl orange (MO) as model dye for azo dyes that are widely used in textile dying, since it possesses an azo group and a sulfonate binding group that could be used to bind to the fabric [21]. An insight into the mechanism of filtration of MO on sodalite (SOM) and faujasite (FAM) clay/zeolite composite membrane is provided herein. MO penetrates the membranes vertically by using its sulfonate group to interact with the positively charged surface of the membrane at pH lower than their points of zero charges and higher than the pK_a_ of MO.

## 2. Materials and Methods

### 2.1. Chemicals and Reagents

Hydrochloric acid (HCl, 37%), sodium silicate (Na_2_Si_3_O_7_), sodium aluminate (NaAlO₂), sodium orthosilicate (Na₄SiO₄), sodium hydroxide (NaOH, 99%), and methyl orange (C_14_H_14_N_3_O_3_S.Na, 95–98%) are obtained from Somaprol Chemicals and Laboratory Reagent Ltd., Casablanca-Morocco. Millipore deionized water (resistivity = 17.2 MΩ.cm) is used for all the experiments. Polyethylene bottles are used in the preparation of the precursor mixtures to avoid contamination with silicon from the glassware.

### 2.2. Preparation of Clay Supports

The clay used in this study is sampled from, Midelt, in the high plains between the Middle Atlas and High Atlas region of Morocco. The clay is crashed to fine particles, then the powder is sifted on AFNOR standardized sieves. The powder with granulometry Φ ≤ 63 µm, 63 µm ≤ Φ ≤ 160 µm and 160 µm ≤ Φ ≤ 250 µm are used for the preparation of the membrane supports. A sample of 3.88 g of the clay powder is thoroughly mixed manually with 0.12 g of activated carbon (3% *w/w* of the clay powder mass) for a period of 10 min, then the mixture is inserted into a stainless die and uniaxially pressed under a pressure of 10 tons’ load (780.8 bar) to obtain raw pellets (flat discs) of 4.0 cm in diameter and 2.0 mm in thickness. The obtained discs are sintered to 1000 °C in a muffle furnace (type NABER 2804) following a suitable heating program, which is developed based on thermal transitions happening in the clay studied previously by our group [13,22].

### 2.3. Preparation of the Clay/Zeolite Composite Membranes

#### 2.3.1. Synthesis of Sodalite Zeolite 

The sodalite is synthesized from two precursors; the aluminate precursor is prepared from 0.075 mol NaOH, 7.15 × 10^−4^ mol NaAlO_3_, 0.277 mole H_2_O and the silicate precursor which is prepared from 0.027 mole, NaO_3_Si·9H_2_O, 0.389 mole H_2_O. The two mixtures are stirred in sealed polypropylene bottles and are left to mature for 24 h at room temperature. The aluminate precursor is added to the silicate precursor dropwise under stirring for 10 min, then the flask is placed in the oven at T = 80 °C for 24 h. The precipitate is filtered and washed several times with deionized water until a pH of the filtrate reach 10. The obtained solid is dried overnight at 80 °C in the oven and kept for further analysis [23,24].

#### 2.3.2. Synthesis of Faujasite Zeolite

The germination gel is prepared by mixing 0.0255 mole of NaOH, and 5.328 × 10^−3^ mole of NaAlO_3_ in 0.277 mole of H_2_O, then 0.02 mole of NaO_3_Si·9H_2_O are added to the mixture under vigorous stirring. The resulting mixture is sealed in a polypropylene container and is left to mature for 24 h at room temperature and under vigorous stirring. While the growth gel is prepared by dissolving 8.75 × 10^−4^ mole of NaOH and 0.033 mole of NaAlO_3_ in 1.82 mole of H_2_O. After the dissolution, 0.125 mole of NaO_3_Si·9H_2_O is added gradually to the mixture under vigorous stirring. The gel is kept to maturity for 2 h. The germination gel is added under vigorous stirring to the growth gel. Then, the obtained mixture is placed in the oven at 80 °C for 24 h in a sealed polypropylene container. The obtained solid is filtered and washed several times with deionized water until the pH of the filtrate reaches 10. Then, the solid is dried overnight in the oven at 80 °C and kept aside for further analysis [25].

#### 2.3.3. Sodalite (SOM) and Faujasite (FAM) Composite Membranes’ Preparation

The preparation of SOM and FAM membranes is carried out using the hydrothermal method, which is described in a previous study [25]. Sintered flat disc supports at 1000 °C (40 mm × 2.0 mm) are introduced in a horizontal position in the lined Teflon autoclave housed in a stainless steel jacket containing the mature precursors described in the previous paragraph. The autoclave is placed overnight in the oven at a temperature of 80 °C.

### 2.4. Characterization Techniques

The clay and zeolite synthesized materials are characterized at different stages; as raw material for the preparation of the clay supports, as consolidated sintered supports, as zeolite powders, and as composite membranes (SOM and FAM). Different analysis techniques are used for the physical/chemical and morphological characterization of the clay and zeolite materials. Fourier Transform Infrared Spectroscopy (FTIR) is carried out by dispersing material powders in KBr dried at 105 °C and are recorded on Fourier Transform Spectrometer Type JASCO-4000 in 4000–400 cm^−1^ the range [26]. X-ray diffraction patterns (XRD) of the clay and zeolite materials are obtained using X-ray diffraction (XRD) powder diffractometer type X’PERT MPD-PRO equipped with a diffracted beam monochromator and Ni filtered CuK_α_ radiation source (λ = 1. 5418 Å). The patterns are recorded using a wide-angle in the range between 2θ = 2° and 50° at a step of 0.02° and a scan rate of 2 s/step [22,27]. The morphology of the SOM and FAM membranes and the supports on which they are deposited have been performed using scanning electron microscopy (SEM) type Topcon EM200B, equipped with an energy dispersive X-ray (EDX) analysis, to detect the elemental analysis of micrographs scanned by the SEM. Thermogravimetric analysis is performed using a thermal analyzer type Microbalance 2960 SDT V 3.0. The samples are heated linearly in the air from a room temperature of 21 °C to 800 °C, with a heating speed of 1.0 °C/min. The importance of thermogravimetric analysis is that it gives an idea about thermal phenomena happening in the clay under heating. Results of the analysis are given in Figure S1 and Table S1 (Supplementary Materials), these data help in planning an appropriate heating program to sinter the clay supports up to 1000 °C. Nitrogen adsorption/desorption measurements are carried out using Micromeritics ASAP 2010 to calculate the textural parameters, namely specific surface S_BET_(m^2^/g), the total volume of the pores (V_t_, cm^3^), pores’ diameter (D (Å)) and pores’ size distribution (PSD). These parameters are computed from Brunauer–Emmet–Teller (BET) theory and the BJH algorithm for pores’ diameters between 17 and 3000 Å [22]. The point of zero charge is determined by potentiometric titration according to the procedure that is reported by Dehmani et al. [26].

### 2.5. Filtration Tests and MO Concentration Measurements

**Figure 1:**
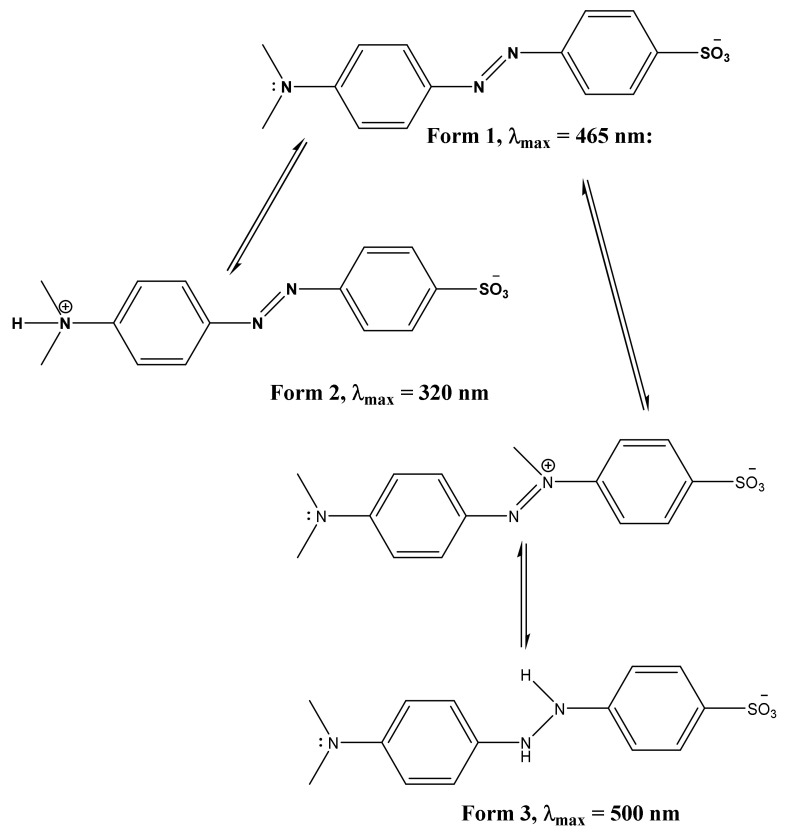
The species formed after protonation of methyl orange.

The MO filtration experiments are performed at a constant temperature of 25 °C and natural pH (without fixing), which corresponds to the natural conditions of industrial discharges. Samples of 10^−4^ M solutions of MO are prepared in deionized water with a pH = 5.5. Usually, MO adopts two forms; in basic medium, it adopts Form 1 while in an acidic medium, the dimethylamino group undergoes a protonation to adopt Form 2, while the protonation of the azo group results in Form 3 (Figure 1) [28,29].

### 2.6. The Filtration Flow Loop

**Figure 2:**
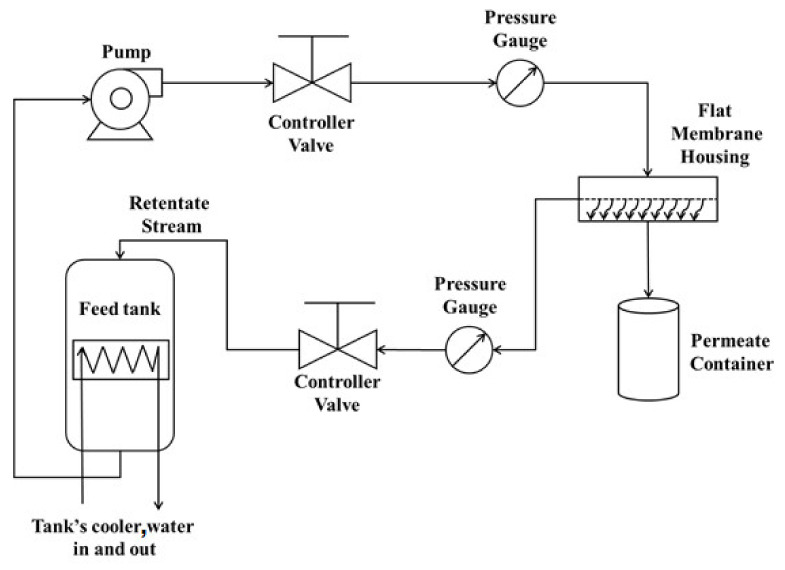
Schematic representation of the frontal filtration flow loop.

Filtration and water permeation tests are performed on the supports, SOM and FAM composite membranes (clay/zeolite) using a homemade flow loop that is designed and built in our laboratory (Figure 2). The flow loop consists of a feed-in container, a circulation pump, control valves, pressure gauges to regulate the filtration rate, and the housing of the flat disc membrane with three entries to allow for a cross-flow filtration. The high-pressure pump provides the dye contaminated wastewater at a constant pressure of 0.5 bar. The feedwater is split into a filtered clean water solution or a more polluted retentate. Two flow regulation valves are being used; one at the feed-in entry and the other at the retentate exit. Both valves are used to maintain the filtration pressure constant while the filtrate is collected in an open atmosphere on the top of a balance to measure the water filtrate every 30 min. The obtained permeate is stored in the fridge at 4.0 °C for further analysis by UV-Vis spectrometer type Shimadzu UV-1240.

Equation (1) is used to determine the retention of MO on the clay supports, SOM and FAM composite membranes. In Equation (1), C_i_ is the molar concentration of methyl orange in the feedstock solution, C_r_ is its molar concentration in the permeate solution.

(1) $$\mathrm{Retention}\;\%=\frac{C_{i}-C_{r}}{C_{i}}\times 100$$

The filtrate flow rate (J) in L·m^−2^·h^−1^ is measured using Equation (2), where V, is the volume of the permeate, t is the filtration time and A, is the filtration surface of the membrane delimited by the internal O-ring’s diameter and is found to be 7.07 × 10^−4^ m^2^.

(2) $$J=\frac{V}{\left(A\cdot t\right)}$$

## 3. Results and Discussion

### 3.1. X-ray Fluorescence Analysis

**Figure 3:**
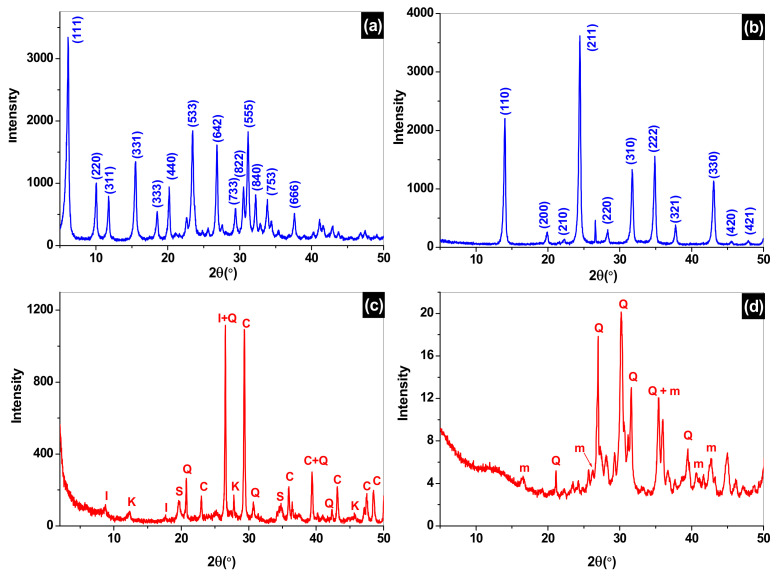
X-ray diffractogram of faujasite (**a**), sodalite (**b**), raw clay (**c**) and clay sintered to 1000 °C (**d**). c: calcite, I: illite, k: kaolinite, m: mullite, Q: quartz, s: smectite.

X-Ray fluorescence analysis of the raw clay material used to prepare the clay supports is represented in Table 1. Silicon, calcium, and aluminum oxides form the major components of the clay with 68.58%. Taking into consideration that kaolinite, calcite, illite, and smectite are the only Si-, Ca- and Al-bearing minerals, whose peaks are observed in the XRD pattern of the clay mineral (Figure 3). It could be concluded, that the contents of those elements are directly related to the presence of kaolinite, illite, smectite and calcite. The iron oxide (Fe_2_O_3_) is present with a 5.22% which is low compared to natural clays. The iron is usually associated with kaolinite, illite and smectite presence, while Ca is mainly related to calcite. Other elements such Mg, K, Ti, and Na are present in XRF with measurable amounts. Mg exist as an interstitial element in illite and smectite, while K could exist in non-exchangeable form where it is held between adjacent tetrahedral of illite. The sum of major oxides Si, Al, Ca, Fe, Mg, K, Ti, and Na in addition to the amount of structural water, are measured by the loss on ignition (LOI), present an average of 99.76%, indicating that the clay contains negligible amounts of those elements (P, Mn, Sr, Zn, and Cr). The loss on ignition (LOI) is found to be 19.5%, which is within the range of 18.0 to 32.1% reported for the clay minerals [30].

### 3.2. X-ray Diffraction Analysis

X-ray diffraction analysis is performed for the two synthesized zeolites, sodalite and faujasite, the raw clay materials which is used for the fabrication of the support and the sintered clay to 1000 °C, which represents the clay support that is used for the synthesis of the composite membranes SOM and FAM.

The X-ray diffraction of sodalite and faujasite show that they are well crystallized and the obtained spectra correspond well to the sought phases compared with those of the standard zeolite given in the literature (collection of simulated powder patterns for zeolites). The crystalline parameters and chemical formula are given in Table 2 [31,32].

The X-ray analysis of the raw clay powder and sintered at 1000 °C are presented in Figure 3c,d, respectively. A mixture of mineral phases such as quartz (Q), calcite (c), kaolinite (k), smectite (s), and illite (I), are identified in the raw clay material by their 2 theta positions [22]. While the X-ray diffraction spectrum of the sintered clay at 1000 °C is presented in Figure 3d and indicates that peaks associated to kaolinite, calcite, illite and smectite phases disappeared, while new peaks are attributed to the meta-kaolin new phase appearance (m, Al_2_O_3_,2SiO_2_) [31].

### 3.3. Adsorption/Desorption of Nitrogen (N_2_) Analysis

**Figure 4:**
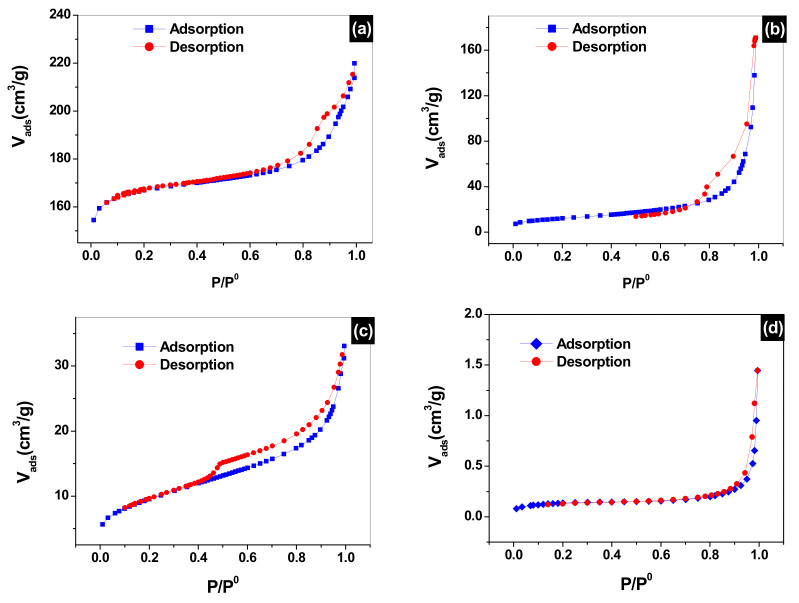
N_2_ adsorption/desorption isotherms of (**a**) faujasite, (**b**) sodalite, (**c**) raw clay mineral and (**d**) 160 µm ≤ Φ ≤ 250 µm support sintered at 1000 °C.

The nitrogen adsorption/desorption study based on BET theory and BJH algorithm of faujasite, sodalite, the clay powder used for the preparation of the supports as well as the 160 µm ≤ Φ ≤ 250 support sintered at 1000 °C, are presented in Figure 4. The obtained isotherms are type IV with a d-loop and type H3 hysteresis according to the IUPAC classification. This indicates that these solids are mesoporous. The obtained specific surfaces are given in Table 3.

The specific surface of the faujasite and sodalite are very large compared to that of the raw clay material and the sintered support. This is due to the zeolite structural ordering, which yields very narrow pore size leading to optimal high surface area [33]. The volume of the pores of the clay powder is 0.04108 cm^3^/g for the clay powder, which is reduced down to 2.13 × 10^−3^ cm^3^/g for the 160 µm ≤ Φ ≤ 250 support under the effect of sintered at 1000 °C, which leads to the fusing of the grains. Deposition of sodalite and faujasite on the membranes increased the volume of the pores to 0.31837 cm^3^/g, which is indicative of the narrowing of the pores of the clay support by sodalite and faujasite. Pores’ diameters follow the same trend they went from 47.7 Å for the raw clay material down to 35.5 Å for the sintered support at 1000 °C because of the grains fusing, then further decreasing down to 22.8 Å when faujasite and sodalite are deposited, which is due to deposition of zeolite inside the pores of the support.

### 3.4. FTIR Analysis

**Figure 5:**
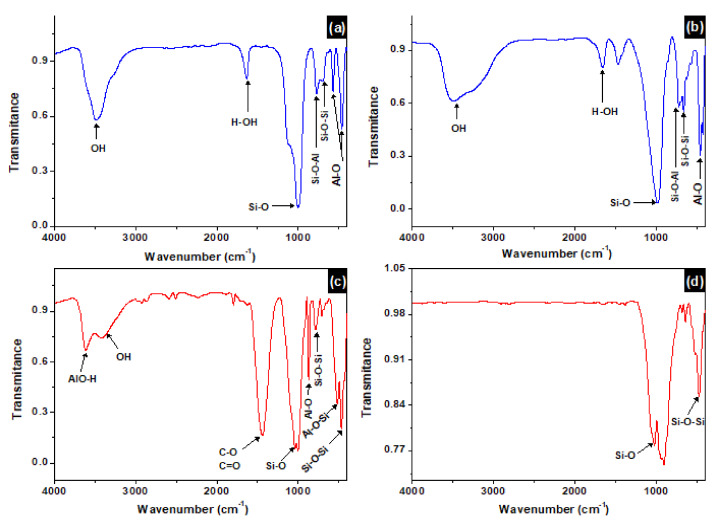
Infrared spectrum of (**a**) faujasite, (**b**) sodalite, (**c**) raw clay material, and (**d**) sintered clay at 1000 °C.

The FTIR analysis of the clay powder and sintered at 1000 °C, as well as sodalite and faujasite zeolite membranes are represented in Figure 5. The FTIR analysis gives the vibrations of the studied materials in the range of 4000 cm^−1^ to 400 cm^−1^. Figure 5a,b presents the spectrum of sodalite and faujasite, respectively. A comparison between the two spectra reveals similarities between the two zeolites. Two bands appearing one at 3477 cm^−1^, and the other at 1640 cm^−1^ are attributed to the stretching and bending vibrations of the hydroxyls (O-H bonds) and adsorbed water molecules (H-O-H bonds). These two vibrations are intense in the faujasite compared to sodalite. The strong vibration between 1000 cm^−1^ and 1100 cm^−1^ is attributed to the Si-O stretching vibration [34]. The two vibrations at 458 cm^−1^ and 553 cm^−1^ are assigned to the Al-O stretching vibration. However, the two vibrations at 687 cm^−1^ and 762 cm^−1^ are attributed to the stretching vibrations of Si-O-Al and Si-O-Si chemical bonds, respectively [35]. From Figure 5c, several bands are being noticed. The two broad bands appearing at 3615 cm^−1^ and 3449 cm^−1^ are attributed to the stretching vibration of the OH group in the clay sheet and of water adsorbed, respectively. A weak absorption band is detected at 1634 cm^−1^ and is attributed to the bending of the structural water molecules of the clay mineral. The 1440 cm^−1^ vibration is due to the presence of carbonates. The bands appearing at 460 cm^−1^ and 1027 cm^−1^ are attributed to the Si-O and Si-O-Si stretching vibrations, respectively. The weak vibrations appearing at 518 cm^−1^ and 867 cm^−1^ are attributed to the Si-O bending and Si-OH stretching vibrations, respectively. The band at 787 cm^−1^ is due to the stretching vibration of the Si-O-Al bond of Kaolinite. Figure 5d represents the FTIR of the sintered clay at 1000 °C. Two important vibrations have disappeared in this spectrum; the first one is the OH groups on the surface of the clay and adsorbed water at 3615 cm^−1^. Such observations are expected to happen at high sintering temperature where a complete dehydroxylation of the clay material is taking place. The second important vibration that is assigned to the carbonate (CO_3_^2−^), which appears at 1430 cm^−1^ in the raw clay. Carbonates usually decompose at 500 °C [36].

### 3.5. Scanning Electron Microscopy Analysis (SEM)

Surface analysis by scanning electron microscopy (SEM) of the clay supports and of the SOM and FAM composite membranes is carried out to obtain information on their morphologies [37].

**Figure 6:**
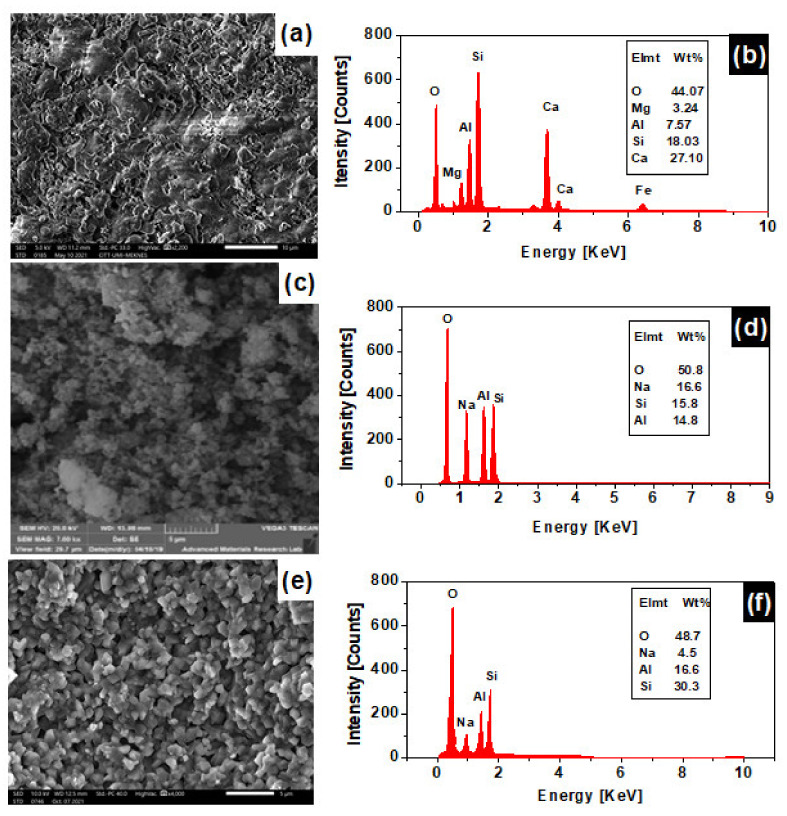
SEM micrographs and EDX analysis of the clay support (**a**,**b**), the SOM composite clay/zeolite membranes (**c**,**d**), and FAM composite clay/zeolite membranes (**e**,**f**).

Figure 6a represents a micrograph of a spray-dried powder of the crashed and powdered clay support. No crystalline structure can be visualized from the micrograph, which is indicative that the clay is being formed from poorly crystalline particles with no defined size. A glassy phenomenon can be assessed from the micrograph and is represented by the fused platelets. This phenomenon happens under the effect of temperature, which helps in the fusing of clay particles to give a support with low porosity. Figure 6b represents the EDX elemental analysis of the clay supports. The support is found to be formed mainly from Si, Al, and Ca (53%). Oxygen contributes by 44% to the elemental contents as a counter cation of the oxide forms of these elements. If oxygen is combined with other elements, 98 wt% compositions is found. The remaining 2 wt% is attributed to other minor elements such as Fe and Ti, which is confirmed by XRF analysis. Figure 6c represents a micrograph of the SOM composite membrane, showing that sodalite crystalizes as fine particles with highly porous grains that aggregate with a cubic crystalline shape. Figure 6d represents the EDX elemental analysis of SOM composite membrane, Na, Si, and Al are the main components of the SOM membrane with an overall wt% of 47.2 wt%. In addition, to 50.8 wt% of oxygen, which complete the oxide form of such elements, bringing the wt% to 98 wt%. The remaining 2 wt% can be attributed to other elements such as carbon. Figure 6e represents an SEM micrograph of the Faujasite crystals grown on the clay support (FAM). It shows the existence of crystals with an average size ranging from 0.53 µm up to 1.8 µm having a bipyramidal shape with a square or Cubo octahedral base characteristic of the Faujasite-type zeolite [24]. Figure 6f represents the EDX elemental analysis of the FAM membrane, Na, Al, and Si composition is 51.4 wt% in addition to oxygen percent of 48.7 wt%, bringing the overall percentage to 100%, which is indicative of the purity and best crystallinity of the faujasite compared to sodalite on the clay supports. The wt% of Na in FAM (4.5 wt%) is found to be lower than the SOM membrane (16.6 wt%).

### 3.6. The Point of Zero Charge of the Clay Supports, SOM and FAM Membranes

The surface charge density, σ_o_ in μC·cm^−2^, is calculated by computing the curve of the blank titration, carried out in the absence of the solid, with that obtained in the presence of the solid. Knowing the specific surface area of the studied solid, the expression of the surface charge density is given by Equation (3).

(3) $$\sigma =\Delta V\cdot \frac{F\cdot C_{B}\cdot 10^{-4}}{{A\cdot m}_{s}}\left(\frac{\mu C}{{\mathrm{cm}}^{2}}\right)$$

where F is Faraday’s constant (96,480 C·mol^−1^), A is the specific surface area of the material studied in m^2^·g^−1^, C_B_ is the concentration of the base used in the titration in mol·L^−1^, m_s_ is the mass of the tested solid in g·L^−1^ and ΔV is the volume variation between the titrated suspension and the blank at the same pH [38].

**Figure 7:**
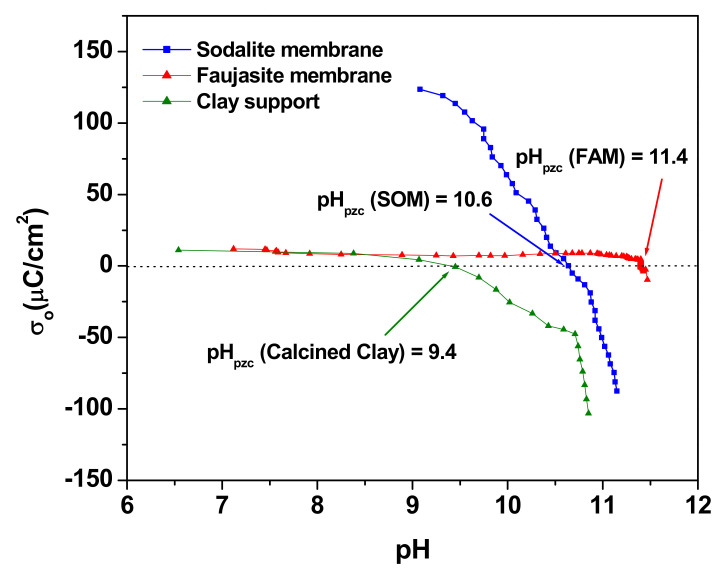
Charge density curves for clay support (olive), SOM composite membrane (blue), and FAM composite membrane (red).

Figure 7 represents the charge density versus pH for the clay support, the SOM, and FAM composite membranes. The three curves cross the *x*-axis at their corresponding point of zero charge (pH_pzc_), which are found to be 9.4, 10.6, and 11.4 for the clay support, the sodalite (SOM), and faujasite membranes (FAM), respectively. Below pH_pzc_, the clay support and zeolites membranes are positively charged, while at higher pH, their surfaces are negatively charged.

### 3.7. Calibration of the Spectrophotometer for Methyl Orange Analysis

For the calibration of the spectrophotometer, the analysis of MO solutions is performed using a series of aqueous solutions containing between 10^−6^ and 10^−4^ M of this pollutant. For all experiments, a wavelength scan is made between 200 and 600 nm. The obtained UV-Visible spectra are presented in Figure S2 (Supplementary Materials). All obtained UV-Vis spectra show two absorption bands; one at λ_max_ = 270 nm presented with very low intensity and corresponds to the aromatic rings and the other is centered around λ_max_ = 465 nm with very strong intensity and corresponds to the azo dyes’ double bond (-N=N), this later band is used for the analysis of MO in water.

### 3.8. Water Flux Permeability for the Clay Supports, SOM, and FAM Membranes

**Figure 8:**
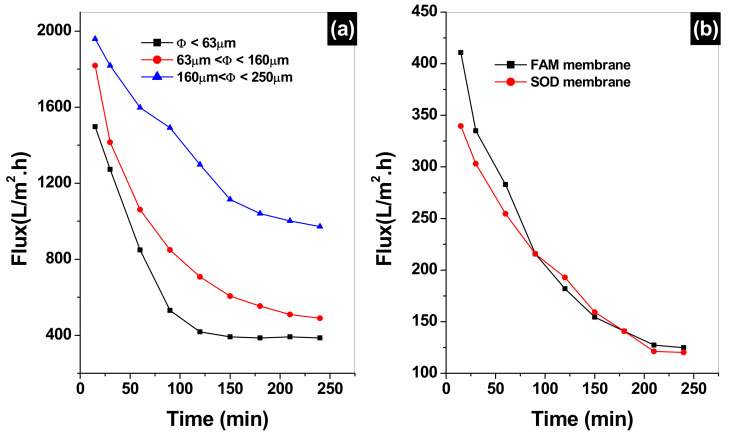
(**a**) Water flux variation versus filtration time for the three supports made from Φ ≤ 63 µm, 63 µm ≤ Φ ≤ 160 µm and 160 µm ≤ Φ ≤ 250 µm granulometries, (**b**) sodalite (SOM) and faujasite (FAM) composite zeolite/clay membranes.

After preparing the clay supports and membranes, preliminary water permeation tests are performed for different particle size supports as shown in Figure 8. The water fluxes of the three supports made from three granulometries Φ ≤ 63 µm, 63 µm ≤ Φ ≤ 160 µm and 160 µm ≤ Φ ≤ 250 µm are represented in Figure 8a. Initial fluxes of 1508, 1817, and 1955 L·m^−2^·h^−1^ are found for the three support made from Φ ≤ 63 µm, 63 µm ≤ Φ ≤ 160 µm and 160 µm ≤ Φ ≤ 250 µm, respectively. These fluxes decrease with the increase of time to minimum fluxes of 388, 555, and 1405 L·m^−2^·h^−1^ for the three supports, respectively, after a working time of 2 h and 90 min. On further increase of the filtration time, the fluxes are stabilized at final values of 384, 500, and 976 L·m^−2^·h^−1^ for the three supports, respectively. This test shows that the two supports with Φ ≤ 63 µm, and 63 µm ≤ Φ ≤ 160 µm granulometries get clogged quickly compared to 160 µm ≤ Φ ≤ 250 µm, which is indicative of the narrow pore size of the two supports. Therefore, the 160 µm ≤ Φ ≤ 250 µm support is chosen for further analysis and the deposition of sodalite (SOM) and faujasite (FAM) membranes. Figure 8b, represents the water fluxes of both membranes SOM and FAM. The fluxes of SOM and FAM membranes decline from initial values of 399 and 411 L·m^−2^·h^−1^ to minimal values of 120 and 129 L·m^−2^·h^−1^, respectively, after a working time of 3 h and 48 min, then get stabilized for the remaining time of the filtration. The equity of the final fluxes of the two membranes (SOM and FAM) is indicative of close pore sizes. However, water fluxes of both membranes are found to be 8 times slower than the 160 µm≤ Φ ≤ 250 µm support. This indicates that the deposition of both zeolites contributes to pore size tuning of the clay support, which can help in efficient removal of the MO dye.

### 3.9. Filtration through 160 µm ≤ Φ ≤ 250 µm Support, SOM, and FAM Composite Zeolite/Clay Membranes

**Figure 9:**
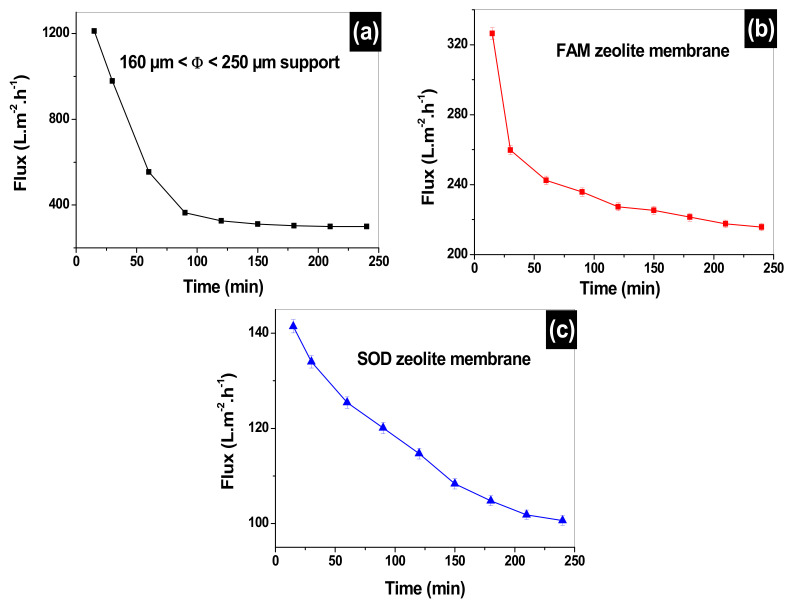
MO flux variation versus filtration time for: the 160 µm ≤ Φ ≤ 250 µm support (**a**), faujasite (FAM) (**b**) and sodalite (SOM) (**c**) composite zeolite/clay membranes.

After the performances of the clay support, SOM and FAM membrane are assessed using deionized water, it is very important to compare the water fluxes to the MO pollutant fluxes. Figure 9 represents the MO fluxes as a function of filtration time. Initial MO fluxes of 1211, 327, and 141 L·m^−2^·h^−1^ are obtained at 15 min filtration time. The support flux declines sharply to 357 L·m^−2^·h^−1^, which makes up 70% of the initial flux if compared to 50% water flux of the same support. Such observation indicates that the support is being presented with wide pores that get clogged quickly with methyl orange. The MO flux on FAM membrane decline to 241 L·m^−2^·h^−1^ after 1-h filtration (26% decline). While the MO flux for the SOM membrane declines slowly to 100 L·m^−2^·h^−1^ after 4 h of filtration (29% decline). These declines in MO fluxes on FAM and SOM are far less than their water fluxes which show a decline of 68%. This is due mainly to the clogging of the pores of the membranes by MO from the starting of the experiment, which leads to slow filtration with comparison to deionized water.

### 3.10. Retention of MO on the 160 µm ≤ Φ ≤ 250 µm Support, SOM, and FAM Composite Zeolite/Clay Membranes

**Figure 10:**
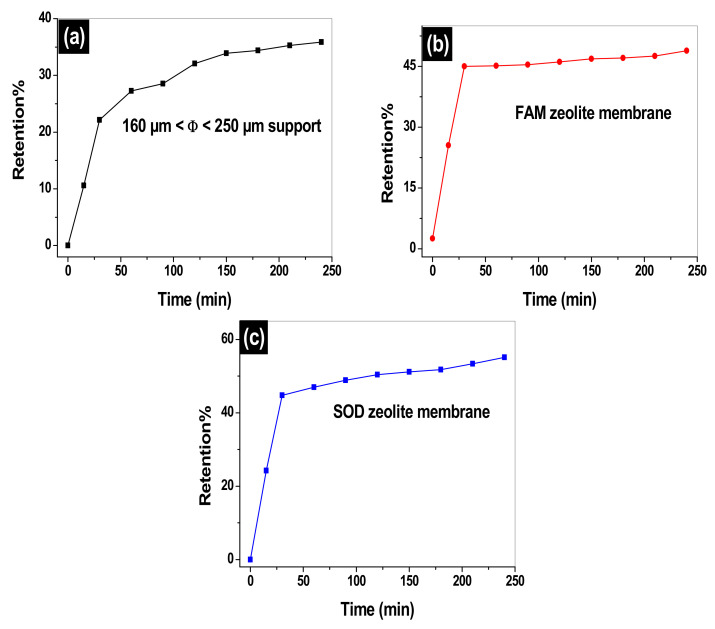
MO percentage of retention versus filtration time on: 160 µm ≤ Φ ≤ 250 µm support (**a**), faujasite (FAM) (**b**) and sodalite (SOM) (**c**) composite zeolite/clay membranes.

Figure 10 shows the retention percentage evolution of MO versus the filtration time. It is observed that the increase in the amount of the MO adsorbed is related to the number of active sites. The MO retention by the support and both membranes (FAM and SOM) is found to vary inversely with the MO fluxes. The retention percent of MO increases sharply from 10% at 15 min to 22% after 28 min filtration on the support. On further increase of filtration time, the retention reaches a maximum value of 35% at 3 h and 48 min as it gets stabilized for the remaining time of the experiment. While the FAM and SOM membranes start both with percentages of retention of 25% at 15 min and continues to increase to final percentages of 45.2% after 30 min of filtration time for both membranes. The retention percent slightly increases to 48% for the FAM for the remaining time of the experiment, while SOM membrane continues to perform well until reaching a 55% retention after 4 h of filtration. The filtration of MO on the clay support, the FAM, and SOM membranes is happening in two stages; the first one is a very fast process characterized by the sharp slope of the curve which is due to largely available sites of interactions on the membranes. While the second stage is a slow process and corresponds to a saturated membrane where the MO molecules are sieved through the membrane based on their hydrated form (1.2 nm) [39]. The size of the pores of both membranes is found to be similar and it is in the order of 22.8 Å (2.28 nm), which is quite large when is compared to MO molecular size. The filtration of MO is not necessarily in its monomeric form, but it can be in its dimeric or aggregated form. Barakat et al. has shown that MO can be aggregated in solution [40].

Size exclusion may be dominant. However, the charge repulsion between the clay support, SOM, and FAM membranes can play an important role in the rejection of the methyl orange. The pH at which MO filtrations are carried out is set between 5.5 and 6.5, which are below the pzc of the clay support, SOM and FAM membrane (pHpzc (clay support) = 9.4, pHpzc (SOM) = 10.6, and pHpzc (FAM) = 11.4). Filtration surfaces are positively charged at the pH of filtration, however, the MO exists in its basic form 1 (Figure 1). The interaction of MO with the positively charged clay support and membranes may have happened through the sulfonate negatively charged group. This leads us to believe that MO penetrates through the membranes using its vertical geometry.

During the filtration using the FAM membrane, the number of MO molecules decrease as an equilibrium state is believed to be reached. Indeed, the adsorbed amount is being stabilized as it reaches a maximum value of around 35% for the filtration of MO by the support, around 45.2% for the FAM, and around 55% for the SOM membrane. The clay support and SOM composite membrane, presented with the highest MO retention were selected for post removal analysis. Figures S3 and S4, (Supplementary Materials), represent, the FTIR before and after filtration of MO. New vibrations at 3440 cm^−1^ and at 1643 cm^−1^ are assessed, these correspond to the stretching vibrations and bending of hydroxyl groups (OH) of the water molecules adsorbed on the outer layer and between the layers of the clay and zeolite materials. This confirms the competitive adsorption between the molecules of the Methyl orange (OM) and those of water. In addition, an increase in the intensity of the bands located at 969 cm^−1^, 1010 cm^−1^,679 cm^−1^,650 cm^−1^, and 480 cm^−1^, which are attributed to the benzene ring of the methyl orange. The band detected at 1436 cm^−1^ corresponds to the vibration of the nitrogen double bond (N=N), while the band that appears at 1202 cm^−1^ is attributed to the S=O stretching vibration of the sulfonate group. The interaction can be carried out between the groups of the clay and zeolite silanol Si-OH^+^ and the sulfonate (${\mathrm{SO}}_{3}^{-}$) of the methyl orange. The SEM micrograph represented in Figure S5 (Supplementary Materials) shows formation of small particles agglomerates of MO on the clay support’s and the SOM composite membrane’s surface. The EDX spectra corresponding to these images demonstrate the appearance of carbon element and an increase in the atomic percentage of oxygen, which confirms the adsorption of methyl orange by these materials. The MO retention of MO 45.2% for FAM and 55% for SOM are found to be comparable to the results found for photocatalytic degradation of MO using nanocomposite membranes. For example, Filice et al. [41] used a hybrid nanocomposite Nafion membrane containing TiO_2_ nanoparticles, and graphene oxide (GO) for the removal of MO under UV/Vis irradiation. Nafion was found to remove 47% of MO, while Nafion-GO removed 46% after a working time of 3 h. However, when Nafion is modified with sulfonate pendent arms (Nafion-GO_SULF_) or TiO_2_ (Nafion-GO_SULF_), the MO removal attain 71% and 67%, respectively. In another study, Li et al. [42] prepared a MOF-NFM membrane by germination of H_3_PW_12_O_40_ @UiO-66 crystals on polyacrylic acid (PAA)-poly (vinyl alcohol) (PVA) nanofibers, and used it for simultaneous photocatalytic removal of MO and formaldhyde. The nanofibrous membrane was found to be efficient in removing MO at 97.4% at working time of 3.0 min, which declined to 46.1% after a working time of 15 min in the absence of formaldehyde (FA), while 97.5% degradation of MO is achieved with the same membrane in the presence of 3.0 mL of FA at working time of 2.0 h. On the other hand, direct filtration of MO on a polytetrafluoroethylene membrane using the vacuum membrane distillation (VMD) was found to surpass the FAM and SOM prepared membranes [43]. More than 99% rejection was achieved by this membrane at various feed temperatures, mass flowrate, dye concentration, and vacuum pressure.

## 4. Conclusions

In this study, we have provided and insight into the capacity of zeolite clay membranes to eliminate one major water pollutant, the methyl orange dye, by filtration on newly synthesized sodalite and faujasite zeolite membranes deposited on mesoporous clay support prepared by the powder metallurgy technique. The hydrothermal method is used to synthesize the SOM and FAM membranes. The results show that, by using a customized flow loop, a retention percentage of 35%, 45.2%, and 55% for 160 µm ≤ Φ ≤ 250 µm support, faujasite, and sodalite clay/zeolite composite membranes, respectively. Therefore, the filtration method on the clay membranes based on zeolite faujasite and sodalite can be used as an alternative ecological method to remove dyes and other toxic chemicals from wastewater. The retention percent were comparable to those found for photocatalytic degradation of MO using nanocomposite membranes, while polytetrafluoroethylene membrane direct filtration of MO surpasses the FAM and SOM prepared membranes. Hence, subsequent studies are necessary to determine the effect of factors such as pH, temperature, flow, additive substrates on membrane which will lead to an improvement in the retention percentage of the dyes and deepens our knowledge on membrane fabrications and manufacturing.

## Figures and Tables

**Table 1:** Chemical composition of the raw clay.

%Oxide	SiO_2_	Al_2_O_3_	CaO	Fe_2_O_3_	MgO	K_2_O	TiO_2_	Na_2_O	P_2_O_5_	Mn_2_O_3_	SrO	ZnO	Cr_2_O_3_	LOI
Raw Clay	39.33	10.44	18.79	5.22	3.11	2.52	0.62	0.23	0.09	0.07	0.05	0.01	0.01	19.5

**Table 2:** Crystal parameters of the synthesized zeolites.

Materials	Parameters	Crystalline System	Chemical Formula
Faujasite Zeolite	a = b = c = 24.79 A α = β = ϫ = 90°	cubic	(Na_2_)_3.5_[Al_7_Si_17_O_48_]·32 (H_2_O)
Sodalite Zeolite	a = b = c = 8.84 Å α = β = γ = 90°	Square bipyramidale	Na_6_(H_2_0)_8_[Si_6_Al_6_O_24_]

**Table 3:** Structural parameters of adsorption/desorption isotherms of N_2_ on Raw Clay, FAM, and SOM clay/zeolite composite membranes.

Materials	S_BET_ (m^2^/g)	Vp (cm^3^/g)	dp (Å)
Raw clay	34.40	0.04108	47.77
160 µm ≤ Φ ≤ 250 Support at 1000 °C	0.481	2.13 × 10^−3^	35.5
Faujasite	558.75	0.31837	22.80
Sodalite	43.34	0.31837	22.79

## Data Availability

Not applicable.

## Supplementary Materials

The following are available online at https://www.mdpi.com/article/10.3390/membranes12010012/s1, Figure S1: Thermogravimetric analysis (TGA, (a)) and differential thermal analysis (DTA, (b)) of clay powder used in the preparation clay supports, Figure S2: UV-Visible spectra of the standard solutions of methyl orange, inset the calibration curve, Figure S3: Infrared spectrum of the 160 µm ≤ Φ ≤ 250 µm support before and after the filtration of the methyl orange, Figure S4: The infrared spectra for the two membranes (a) FAM and (b) SOM membranes before and after filtration of methyl orange (MO), Figure S5: SEM micrographs and EDS analysis of: the clay support (a,b) and composite SOM clay/zeolite (c,d) after filtration.
