# Supplementary material for: Preparation of Sodalite and Faujasite Clay Composite Membranes and Their Utilization in the Decontamination of Dye Effluents
Source: Membranes (Basel). 2021 Dec 23;12(1):12. doi: 10.3390/membranes12010012 (PMC8782013; doi:10.3390/membranes12010012)
Supplement: Supplementary file 1 [file membranes-12-00012-s001.zip › membranes-1511997-supplementary.pdf]

Preparation of Sodalite and Faujasite Clay Composite Membranes and Their Utilization in the Decontamination of Dye Effluents

Abderrazek El-kordy [1], Abdelaziz Elgamouz [2,*], El Mokhtar Lemdek [1], Najib Tijani [1], Salman S. Alharthi [3], Abdel-Nasser Kawde [2] and Ihsan Shehadi [2]

1 Laboratory of Materials, Membranes, and Nanotechnology, Department of Chemistry, Faculty of Sciences, Moulay Ismail University, PB 11201, Zitoune, Meknes P. O. Box 11201, Morocco; abderrazekelkordy@gmail.com (A.E.-k.); lemdek@gmail.com (E.M.L.); najibtij@gmail.com (N.T.)

2 Pure and Applied Chemistry Group, Department of Chemistry, College of Sciences, University of Sharjah, Sharjah P.O. Box 27272, United Arab Emirates; akawde@sharjah.ac.ae (A.-N.K.); ishehadi@sharjah.ac.ae (I.S.)

3 Department of Chemistry, College of Science, Taif University, P.O. Box 11099, Taif 21944, Saudi Arabia; s.a.alharthi@tu.edu.sa

* Correspondence: aelgamouz@sharjah.ac.ae; Tel.: +971-6516-6769

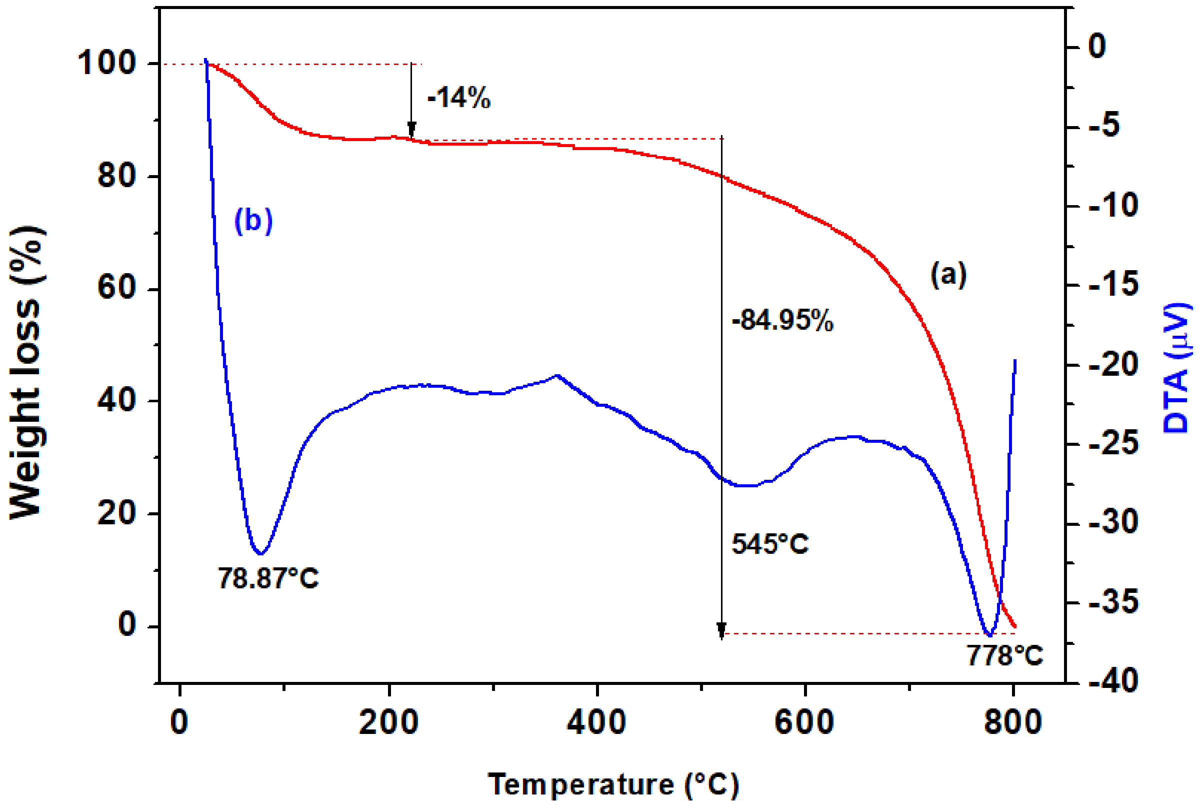

Figure. S1 Thermogravimetric analysis (TGA, (a)) and differential thermal analysis (DTA, (b)) of clay powder used in the preparation clay supports.

Table S1. TGA/DTA peaks attributions for the clay material used in the fabrication of clay supports.

TGA weight Loss (%)	TGA temperature range	DTA peak	DTA peak type	Attribution
14%	28-250°C	78°C	Endothermic	The departure of the water adsorbed on the surface
85%	350-780°C	545°C	Endothermic	Dehydroxylation of illite and kaolinite
	658-800°C	778°C	Endothermic	Decomposition of carbonates
	290-400°C	360°C	Exothermic	Combustion of organic matter

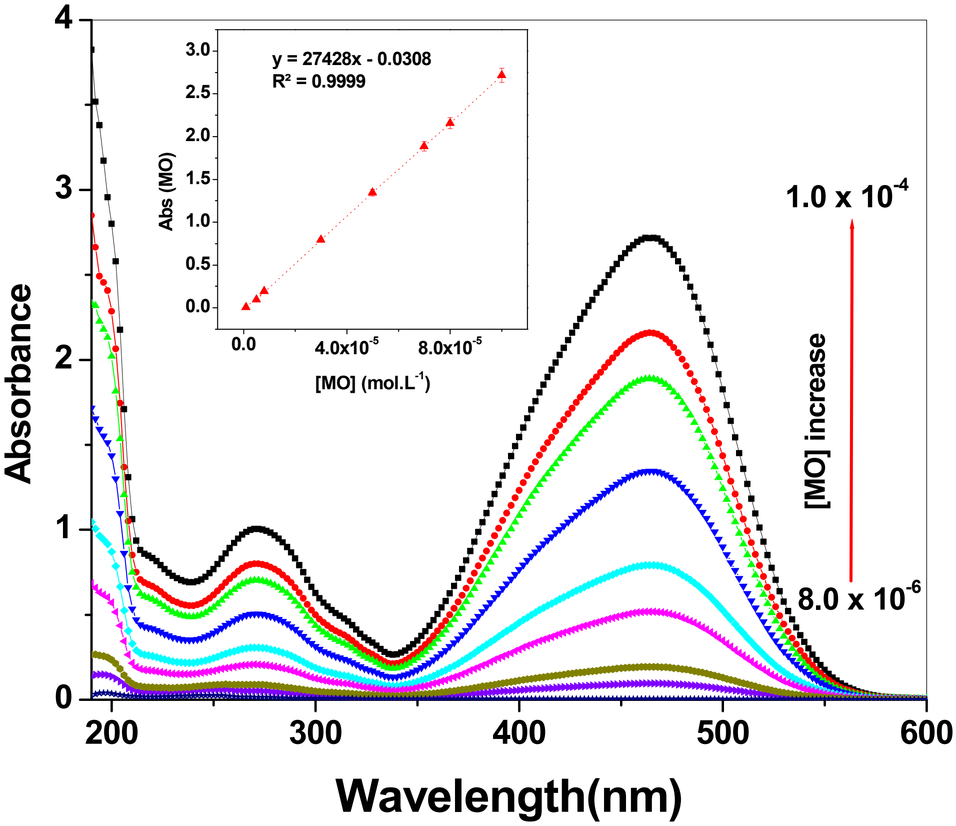

Figure. S2 UV-Visible spectra of the standard solutions of methyl orange, inset the calibration curve.

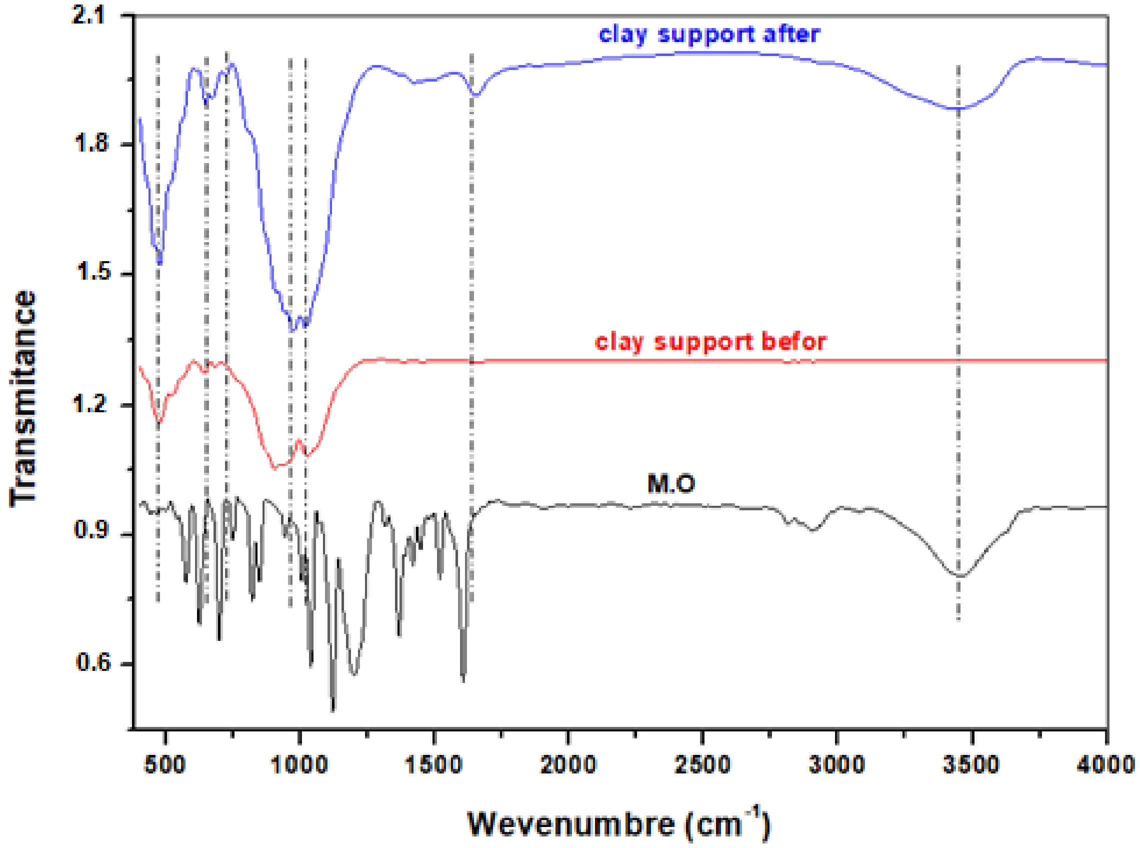

Figure. S3 Infrared spectrum of the 160 μm ≤ Φ ≤ 250 μm support before and after the filtration of the methyl orange.

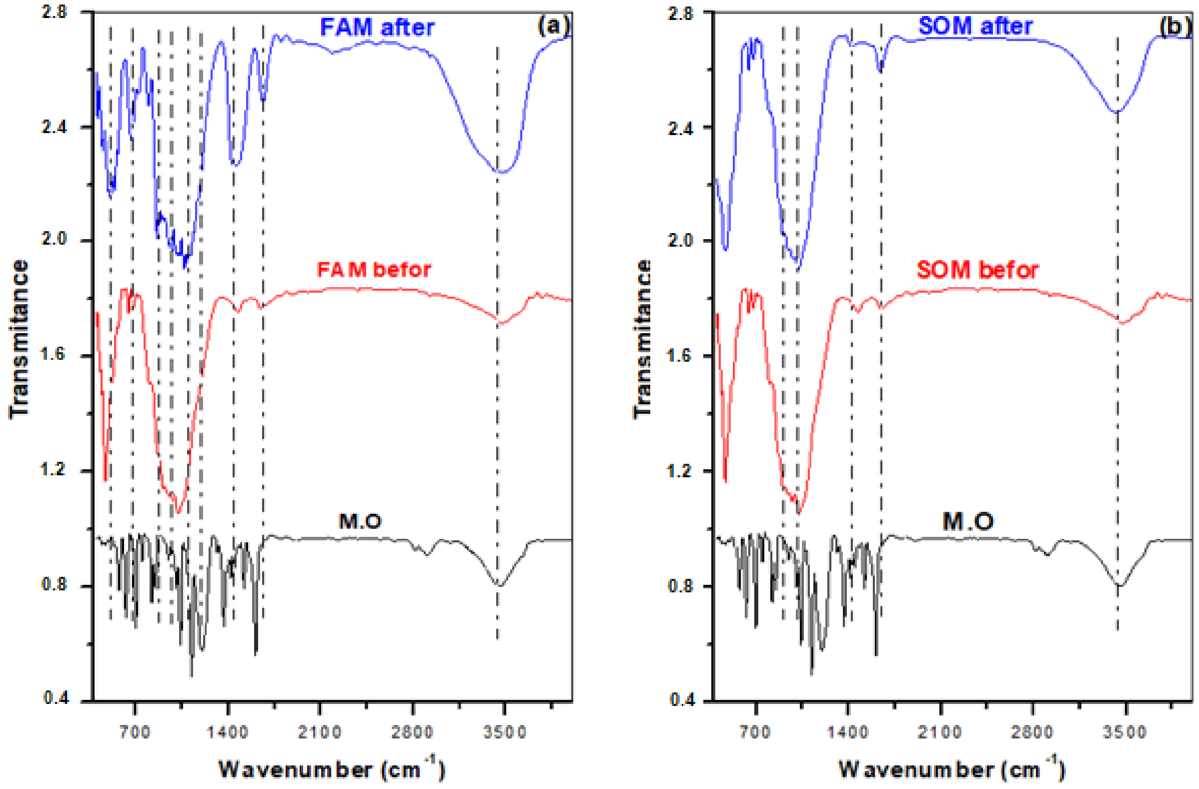

Figure S4 The infrared spectra for the two membranes (a) FAM and (b) SOM membranes before and after filtration of methyl orange (MO).

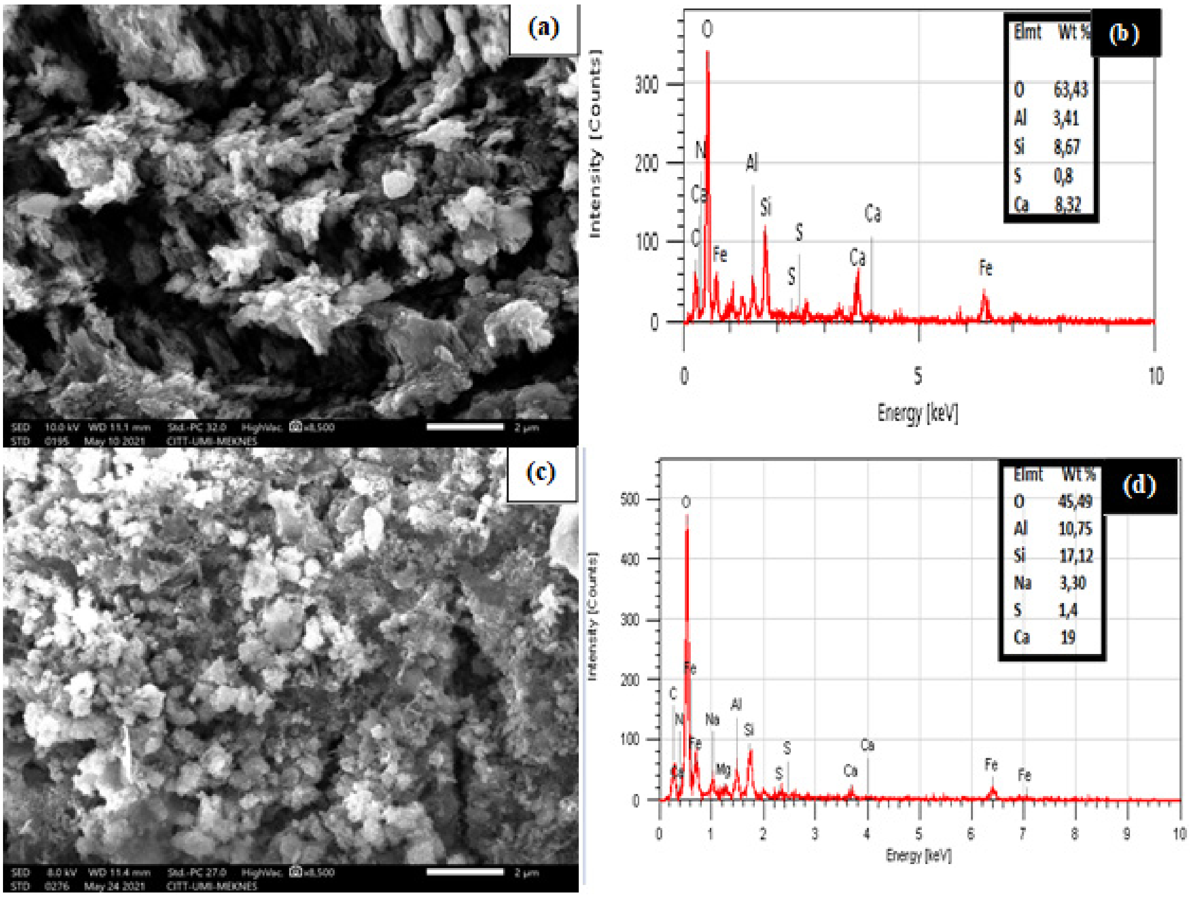

Figure. S5 SEM micrographs and EDS analysis of: the clay support (a,b) and composite SOM clay/zeolithe (c,d) after filtration.
